# Patients’ and parents’ perspective on the implementation of Patient Reported Outcome Measures in pediatric clinical practice using the KLIK PROM portal

**DOI:** 10.1007/s11136-021-02950-x

**Published:** 2021-07-29

**Authors:** Maud M. van Muilekom, Lorynn Teela, Hedy A. van Oers, Johannes B. van Goudoever, Martha A. Grootenhuis, Lotte Haverman

**Affiliations:** 1 Child and Adolescent Psychiatry & Psychosocial Care, Amsterdam Reproduction and Development, Amsterdam Public Health, Amsterdam UMC, University of Amsterdam, Emma Children’s Hospital, Meibergdreef 9, Amsterdam, The Netherlands; 2 Department of Pediatrics, Amsterdam UMC, University of Amsterdam, Vrije Universiteit Amsterdam, Emma Children’s Hospital, Meibergdreef 9, Amsterdam, The Netherlands; 3 Princess Máxima Center for Pediatric Oncology, Utrecht, The Netherlands; 4 Child and Adolescent Psychiatry & Psychosocial Care, G8-136, Amsterdam UMC, University of Amsterdam, Emma Children’s Hospital, Amsterdam, The Netherlands 22660, 1100 DD

**Keywords:** Patient Reported Outcomes, Questionnaires, Patient engagement, Pediatrics

## Abstract

**Introduction:**

The KLIK Patient Reported Outcome Measures (PROM) portal (www.hetklikt.nu) has been implemented since 2011 in clinical practice in over 20 Dutch hospitals. Patients and/or parents complete PROMs before the outpatient consultation and answers are subsequently discussed by clinicians during consultation. This study aims to provide insight into patients’ and parents’ perspective on the use of the KLIK PROM portal in order to optimize its implementation in pediatric clinical practice.

**Methods:**

Patients (12–19 years) and parents (of children 0–19 years) from the Emma Children’s Hospital were invited to participate. A mixed-method design was used; (1) Focus groups were held and analyzed using thematic analysis in psychology, (2) a questionnaire was sent out and analyzed using descriptive statistics.

**Results:**

(1) Eight patients and 17 parents participated. Patients mentioned that KLIK has an attractive layout. However, PROMs were sometimes considered irrelevant and repetitive. Parents valued that KLIK provides insight into their child’s functioning, but they were not satisfied with the extent to which PROMs were discussed by clinicians. (2) 31 patients and 130 parents completed the questionnaire. Overall, patients and parents reported a satisfaction score of 7.9/10 and 7.3/10, respectively. 81% of patients and 74% of parents indicated that KLIK is easy to use.

**Conclusion:**

Patients and parents are generally satisfied with KLIK, however, points of improvement were mentioned. These are currently being addressed by e.g., upgrading the KLIK website, implementing PROMIS item banks in KLIK to reduce irrelevancy and repetitiveness of PROMs, and implementation strategies to improve the discussion-rate. In this way, implementation of the KLIK PROM portal can be further optimized, with the ultimate goal to improve quality of care.

## Introduction

Patient Reported Outcome Measures (PROMs) are increasingly used to monitor and discuss symptoms, Health-Related Quality of Life (HRQOL) and psychosocial functioning of patients in the consultation room with the ultimate goal to enable shared-decision making and patient-centered care [1–3]. Using PROMs in clinical practice has been shown valuable, as it results in more awareness for and increased discussion of patient concerns, higher patient satisfaction, better communication between patient and clinician, and improved treatment outcomes [4–9].

A system that facilitates the use of PROMs in clinical practice is the evidence-based KLIK PROM portal (www.hetklikt.nu) [10–13], which has been implemented in over 20 hospitals in the Netherlands since 2011 [14]. With KLIK, pediatric patients and/or their parents, and adult patients complete PROMs before the outpatient consultation. Answers are converted into an electronic KLIK PROfile (KLIK ePROfile) which the clinician discusses with patients and parents during the consultation [14]. The most important stakeholders in the development and implementation process of the KLIK PROM portal are the users; clinicians as well as patients/parents. From the onset of KLIK, clinicians’ opinions were asked during these processes. For example, clinicians’ preferences for PROM feedback options in the KLIK ePROfile were studied [10], clinicians were involved in the selection of PROs and PROMs for their disease group, and they were consulted in annual evaluation meetings to identify and overcome barriers in the implementation process [14]. Two studies were performed to gain more insight into the experiences of clinicians with KLIK and to identify barriers in the implementation process, with the goal to improve the KLIK PROM portal according to their needs [15, 16]. However, the opinion of the other stakeholder, patient/parents, is also important [17], as engaging patients in KLIK could result in higher patient satisfaction and higher enrollment rates [18–21].

Worldwide, patients are increasingly engaged in PROM development (e.g., item development, comprehensibility) [22] and PROM visualization to patients and clinicians [23]. However, the experiences of patients regarding the use of PROMs in daily clinical practice has received less consideration [24–31]. Available studies explored the experiences of adult patients regarding the use of PROMs in daily clinical practice. Both positive (e.g., improved communication, insight into patient’s functioning, and increased awareness of psychosocial problems) [25, 26, 28–31] and negative experiences (e.g., negative and irrelevant questions in PROMs, unclear purpose of using PROMs) [25–27] were identified. To our knowledge, no studies have been performed focusing on the experiences of pediatric patients and their parents with using PROMs in daily clinical practice. To be able to optimize and further implement the KLIK PROM portal, it is also necessary to gain understanding of their wishes and needs. Therefore, the aim of this study is to provide more insight into the perspective of patients and parents on the implementation of PROMs in pediatric clinical practice using the KLIK PROM portal.

## Methods

### KLIK workflow

**Fig. 1:**
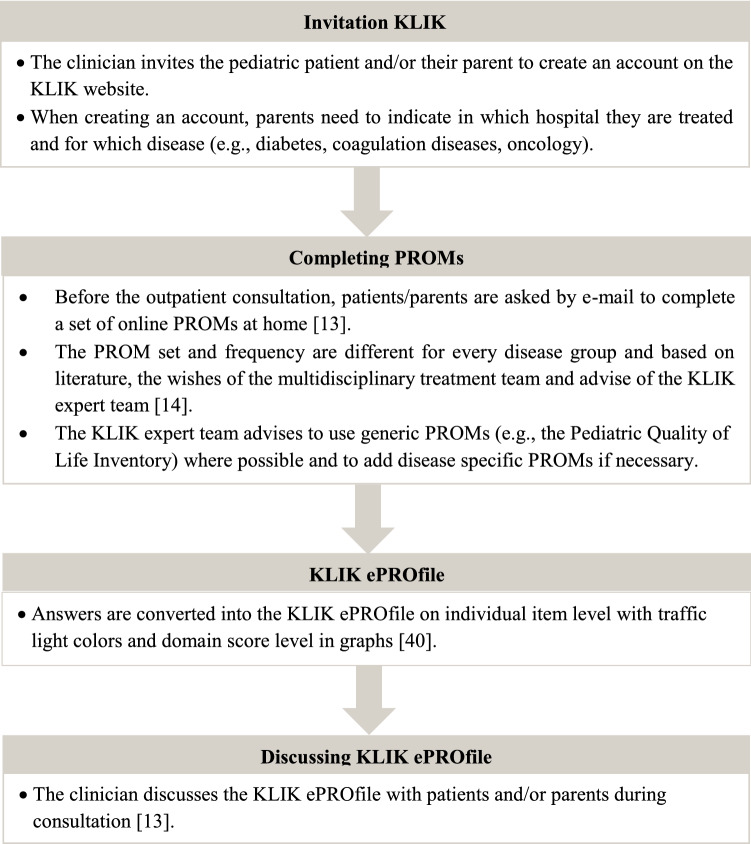
Patient journey of patients and parents using the KLIK PROM portal

The KLIK workflow for pediatric patients and parents consists of several steps; (1) creation of a KLIK account by patients/parents, (2) completion of PROMs by patients/parents before the outpatient consultation, (3) conversion of answers into a KLIK ePROfile, and (4) discussion of the KLIK ePROfile by the clinician during consultation (Fig. 1).

### Design

This study is part of a larger participation study where KLIK users’ (patients/parents) opinion was asked about several aspects of health care and the use of the KLIK PROM portal. This sub-study reports on the evaluation of the KLIK PROM portal. A mixed-method design was used where qualitative and quantitative methodologies were combined: (1) focus groups were held with patients and parents and (2) an evaluation questionnaire was sent out to pediatric patients and parents. The Medical Ethics Committee of the Amsterdam University Medical Centers (Amsterdam UMC—AMC) approved this study. All participants provided informed consent.

### Participants

Patients (12–19 years) and parents (of children 0–19 years) who consult a pediatric department of the Emma Children’s Hospital Amsterdam UMC that uses KLIK as standard part of care, completed KLIK PROMs at least once (questionnaire) or twice (focus groups), and were part of the ‘KLIK panel’ could participate in this mixed-method study. Patients with any chronic health condition could participate in this study as the workflow of the KLIK PROM portal is similar for all patient groups. The ‘KLIK panel’ consists of patients and parents that indicated, during registration on the KLIK PROM portal, that they give permission to be invited for research projects. Eligible patients/parents were invited by e-mail to take part in the focus groups (March 2018) and/or to complete the evaluation questionnaire (June–December 2019). Socio-demographics (age and gender child), information on chronic health condition of the child and years of using KLIK were obtained from the KLIK PROM portal. All participants received a gift card of 5 euros (focus groups) or 10 euros (questionnaire) after participation.

### Procedure

#### Focus groups

Focus groups with patients and parents were held separately and for each focus group inclusion of three to six participants was pursued [32]. Focus groups consisted of a group discussion guided by two moderators (MvM, LT, HvO, or LH). At the start of the focus group, the aim of the study was explained and a short recapitulation of KLIK was provided. Then, to obtain patients’ and parents’ opinion about KLIK, positive and negative experiences with KLIK were discussed using the evaluation technique ‘Complain and Cheer wall’ [33]. Participants were asked to write down their positive experiences on a flip over at one side of the room, what we called the ‘Cheer wall’, and points of improvement on another flip over at the other side of the room, the ‘Complain wall’. Thereafter a group discussion took place and topics on the walls were grouped together into main themes. Duration of each focus group was 60 min. All focus groups were audio recorded.

#### Questionnaire

The questionnaire (separate version for patients and parents, with minor differences regarding language use—Supplement 1) was developed by five researchers of the KLIK expert team and reviewed by five other researchers and one psychologist. Both versions of the questionnaire consisted of 17 closed questions (response options: three- and five-point Likert Scales and Visual Analogue Scales (VAS)) and two mandatory open questions (advantages and disadvantages of KLIK), regarding (1) overall satisfaction with the KLIK PROM portal, (2) completion of PROMs in the KLIK PROM portal, (3) discussing PROMs with the clinician, (4) influence of KLIK on the (preparation of) the consultation, (5) usability of the KLIK PROM portal, and (6) content of PROMs. For three closed questions, an additional mandatory open question was provided, asking about the reason for their answer.

### Analyses

Descriptive analyses were performed using the Statistical Package for Social Sciences (SPSS) version 25.0 to characterize the participants.

Regarding the focus groups, all audio recordings were transcribed verbatim and the transcripts were analyzed independently by MvM and LT in MAXQDA (2018) following the thematic analysis in psychology [34]: (1) highlighting relevant parts of the manuscript, (2) organizing data into meaningful groups by generating initial codes, (3) collating initial codes into themes, (4) refining themes into main- and subthemes, (5) defining the final themes. Analyses were discussed until consensus was reached on the themes. Data saturation was considered attained when no new themes emerged during the analyses of the focus groups.

Regarding the questionnaire, SPSS was used for descriptive statistics (percentages) to provide insight into the experiences of patients and parents with the use of the KLIK PROM portal. Open questions of the evaluation questionnaires were analyzed qualitatively by MvM and LT. This was done by clustering the answers of both patients and parents into main themes following the thematic analysis in psychology [34].

## Results

### Participants

**Fig. 2:**
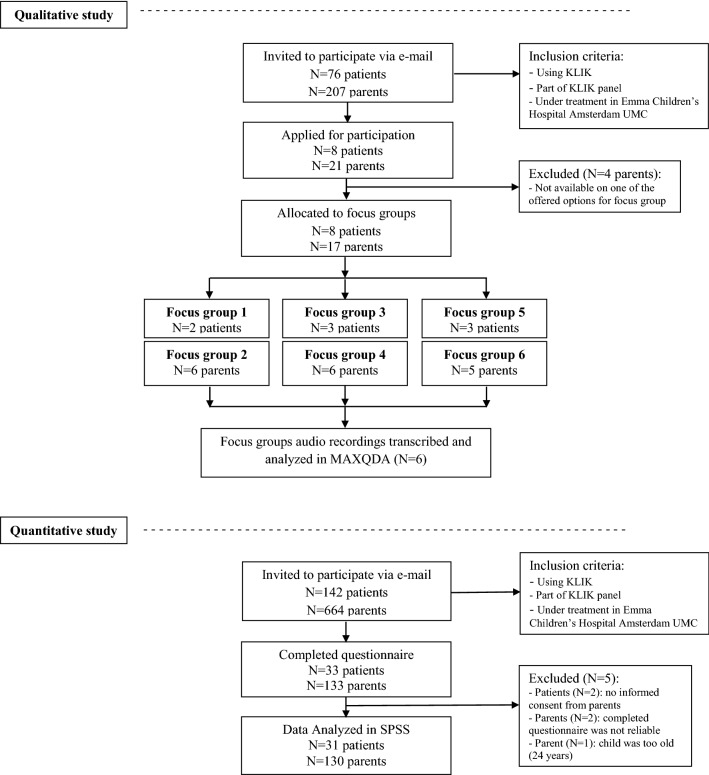
Study and participant flowchart of the qualitative (focus groups) and quantitative study (questionnaire)

Figure 2 shows the study and participant flowchart of this study. In total, 8 patients (three focus groups) and 17 parents (three focus groups) participated in six focus groups. Regarding the questionnaire, 31 patients (response rate: 21.8%) and 130 parents (response rate: 19.6%) participated. One patient and 5 parents participated in the focus groups and completed the questionnaire. Table 1 shows the sociodemographic characteristics of all participants.

**Table 1:** Sociodemographic characteristics of focus group and questionnaire participants

Patients	Focus groups			Questionnaire		
	*N*	*M*	Range	*N*	*M*	Range
KLIK user since (years)	8	3.2	1.1–6.1	31	5.2	1.0–8.2
Age	8	15.3	13.1–18.8	31	15.7	12.4–19.2

		%			%	
Gender (female)	6	75.0		15	48.4	
Chronic health condition						
Juvenile idiopathic arthritis	2	25.0		7	22.6	
Cystic Fibrosis	2	25.0		1	3.2	
Cancer	2	25.0		0	0	
Gastrointestinal diseases	1	12.5		4	12.9	
Home parenteral nutrition	1	12.5		0	0	
Sickle cell disease	0	0		4	12.9	
Other*	0	0		15	48.4	

Parents	*N*	*M*	Range	*N*	*M*	Range
KLIK user since (years)	17	2.8	0.8–6.1	130	3.2	0.3–8.1
Age (of child in KLIK)	17	10.4	2.1–16.9	130	9.3	0.9–19.1

		%			%	
Chronic health condition (child)						
Cancer	6	35.3		0	0	
Juvenile idiopathic arthritis	2	11.8		13	10.0	
Hemophilia	2	11.7		4	3.1	
Home parenteral nutrition	2	11.7		3	2.3	
Gastrointestinal diseases	1	5.9		20	15.4	
Neonatology follow up	0	0		28	21.5	
Other*	4	23.5		62	47.7	

*Only most common conditions groups (> 10% in one of the study groups) are reported, other: cleft lip, endocrinology, nephrology, HIV, dermatology, craniofacial abnormalities, spherocytosis, cystic fibrosis, lysosomal storage disorders, intensive care follow-up, Marfan syndrome, feeding disorders, phenylketonuria, and muscular disorders

### Focus groups

Data saturation was attained as no new themes emerged after analyzing the focus groups. Table 2 (patients) and 3 (parents) depict the most important positive experiences with KLIK and points of improvement for KLIK and corresponding examples of statements. Themes are ranked based on the number of times mentioned (most often to fewest times) by patients and parents during the focus groups.

**Table 2:** Positive experiences and points of improvement mentioned by patients (N = 8) in the focus groups (ranked from most often to fewest times mentioned)

Themes	Positive experiences	Points of improvement
Content of PROMs	‘The questions are clear, recognizable and easy to answer’	‘There is a lot of repetition in questions’
	‘All topics are covered in the questionnaires, not only topics about your disease’	‘The questions are not relevant for every patient and sometimes questions are difficult to understand’
		‘It would be good if questions were administered based on previous answers’
Completion time PROMs	‘Completing the questionnaires does not take too much time’	‘Completing the questionnaires takes a lot of time’
Layout	‘The KLIK website looks nice with the colors that are used’	
	‘Nice that you can see a picture of your doctor’	
Discussion by clinician	‘The answers in the KLIK ePROfile are discussed by the clinician’	‘The clinician often does not discuss the KLIK ePROfile’
		‘Sometimes the clinician does not ask more questions based on my answers’
Insight patients’ functioning	‘By completing the questionnaires you see how you are doing’	
	‘It is good that parents know what is going on’	
	‘With KLIK, clinicians know how you are doing’	
Conversation content	‘With KLIK, not only physical health, but also mental health is discussed’ ‘It helps in discussing topics that you would otherwise not think about’	
Preparation of consultation	‘Completing the questions before the appointment helps you to come up with topics you want to discuss during the consultation’	‘Completing KLIK questionnaires does not help you in preparing for the consultation, it is just something you need to do’
Motivation child		‘I think it is not always necessary to complete the KLIK questionnaires’
		‘I sometimes just do not want to talk about the KLIK topics’
Consultation efficiency	‘The consultation is more efficient when KLIK is used, as the doctor immediately has an overview of how you are doing’	
Anonimity and security	‘It is good that KLIK is well secured’	
	‘As KLIK PROMs are completed on the computer, it feels more anonymous, which results in completing the PROMs more honestly’	
Ease of use	‘It is nice that the KLIK questionnaires can be completed on the computer at home’	‘You cannot go back to the questionnaire if you completed all questions’

All quotes were translated into English

**Table 3:** Positive experiences and points of improvement mentioned by parents (N = 17) in the focus groups (ranked from most often to fewest times mentioned)

Themes	Positive experiences	Points of improvement
Content of PROMs	‘The questions are easy to understand for children’	‘The questions are sometimes not relevant and confronting for children’
	‘All important topics are covered in the questionnaires’	‘It is annoying that every time the same questions are asked’
		‘There is no attention for brothers, sisters and the family situation’
		‘The questions are difficult to understand for young children. I would suggest to make the questions more visual’
Ease of use	‘KLIK is easy to use and it is nice that you can complete questionnaires online’	‘KLIK should be connected with the EHRs, so appointments are automatically linked’
	‘I like the reminder e-mails that are sent by KLIK’	‘I would like KLIK to be available as an app’
Insight patients’ functioning	‘It is nice that parents have insight into the functioning of their child over time’	
	‘With KLIK the clinician knows what is going on and can follow the child over time’	
Discussion by clinician	‘The clinician takes KLIK seriously and always discusses the answers’	‘The KLIK questionnaires are often not discussed by the clinician’
		‘Especially questionnaires about the functioning of parents are not discussed’
Conversation content	‘KLIK is a conversation tool and provides structure and more depth to the conversation’	‘Our consultation has already a fixed structure, so KLIK does not help with that’
	‘It is nice that with KLIK psychosocial functioning is also taken into account’	
Preparation of consultation	‘KLIK helps to start a conversation with your child or partner about the situation before the consultation’	
	‘KLIK helps to think about how it is going and to prepare questions before the consultation’	
Layout	‘The KLIK website is attractive and looks nice for children’	‘It would be good if smileys were used to make KLIK more attractive’
	‘The layout of KLIK is clear and understandable’	
Completion time PROMs	‘The completion time is manageable and not too long’	‘Too many questions have to be completed’
		‘Before I start completing the questionnaires I would like to see how much time it will take’
Detecting problems	‘With KLIK problems are detected early and your child can be referred for help’	
Value and goal	‘I like that with KLIK there is the possibility to report difficulties’	‘Completing KLIK questionnaires feels not useful when it is going well’
		‘It is not totally clear what is done with your answers and if they can be used against you by the government’

All quotes were translated into English

#### Patients

In all focus groups, patients came up with a broad range of experiences with KLIK, both positive, negative and mixed. Themes that were unanimously rated as positive were that the KLIK website has an attractive *layout* (due to the use of colors and pictures), that KLIK *provides insight* into their daily functioning and that KLIK improves the *conversation content* during the consultation, where a broader range of topics is discussed. Furthermore, patients indicated that the *consultation* is more *efficient* when using KLIK and that they are happy about how *secure* the KLIK website is and how their data remains *anonymous*. There were five themes on which patients disagreed. Some patients rated the *content of PROMs* positively, as they cover all important topics and are clear, while other patients indicated that the questions in the PROMs are difficult to understand, repetitive and not relevant for every patient. In addition, *completion time* was rated by some as good and by others as time-consuming, and the KLIK ePROfile is always *discussed* by the *clinician* according to some patients, but not enough by others. Finally, KLIK helps only some patients in *preparing for the consultation*, and patients were ambiguous about *ease of use* of KLIK. The lack of motivation for completing the KLIK PROMs was only mentioned as a negative experience by some patients.

#### Parents

Parents mentioned many similar experiences with KLIK as patients (Table 3). Themes that were unanimously rated as positive were that KLIK helps in *preparing for the consultation* and *provides insight into the patients’ functioning*, although for some parents this insight was also confronting when many problems were reported. In addition, parents were satisfied that by using KLIK *problems are detected* at an early stage and that support can be provided timely. All other themes were evaluated both positively and negatively. Some parents indicated that they are satisfied with the *content of PROMs*, as all topics are covered and questions are easy to understand, while other parents disagreed and indicated that questions are hard to understand for their child, are confronting and repetitive. Parents also had mixed opinions regarding *ease of use* of KLIK, where some thought completing PROMs online is working great, and others thought this could be improved by developing a KLIK app and linking KLIK to the Electronic Health Records (EHR). Furthermore, *discussion* of the KLIK ePROfile *by clinicians* always happens according to some parents, but not often enough by even more parents. Most parents mentioned that the *conversation content* improves as more and different topics are discussed, while some did not recognize this. *Completion time* is manageable for some, but too long for others and the *layout* of the KLIK website is attractive and child-friendly according to most parents, but could be made more attractive by using visuals according to some parents. Finally, some parents indicated that they do not see the added *value and goal* of KLIK, while others disagreed and indicated that KLIK is of great value to the consultation.

### Questionnaire

#### Overall satisfaction with the KLIK PROM portal

Patients and parents reported an overall satisfaction with the KLIK PROM portal of mean = 7.9 and mean = 7.3, respectively, on a VAS ranging from 0 (not satisfied) to 10 (very satisfied).

#### Completion of PROMs in the KLIK PROM portal

As shown in Table 4, 78% of the patients and 84% of the parents agreed that they know why there are asked to complete PROMs via the KLIK PROM portal. Patients and parents reported that the frequency in which they are asked to complete these PROMs varies from once every three years to more than four times a year. Most patients and parents were satisfied with this frequency. When patients and parents are asked to complete PROMs, the majority indicated that they almost always do this. Reasons for not completing the PROMs were: lack of time, forgot to complete, little change in functioning since the last PROM completion, and no motivation. Patients and parents spent on average 13.8 and 15.2 min on completing the PROMs, respectively. More than 80% of both patients and parents were satisfied with this completion time.

**Table 4:** Scores on the domain ‘completion of PROMs in the KLIK PROM portal’ (patients: *N* = 31, parents: *N* = 130)

			Agree–*N* (%)	Neutral–*N* (%)	Disagree–*N* (%)
I know why I am being asked to complete KLIK PROMs	Patients		24 (78)	1 (3)	6 (19)
	Parents		109 (84)	13 (10)	8 (6)
		**4 times a year–*N*** **(%)**	**2 times a year–*N*** **(%)**	**Yearly–*N*** **(%)**	**Other–*N*** **(%)**
How often are you asked to complete the PROMs in KLIK?	Patients	7 (22)	12 (39)	8 (26)	4 (13)
	Parents	21 (16)	29 (22)	38 (30)	42 (32)
			**Yes–*N*** **(%)**	**No, too often–*N*** **(%)**	**No, too infrequent–*N*** **(%)**
Are you satisfied with this frequency?	Patients		29 (94)	1 (3)	1 (3)
	Parents		111 (85)	11 (9)	8 (6)
			**(Almost) always–*N*** **(%)**	**Sometimes–*N*** (%)	**(Almost) never–*N*** **(%)**
When you are asked to complete the PROMs in KLIK, how often do you do this?how often do you do this?	Patients		28 (90)	3 (10)	–
	Parents		123 (95)	2 (1)	5 (4)
			*M* (range)		
I spend on average.. minutes on completing the KLIK PROMs	Patients		13.8 (5–30)		
	Parents		15.2 (0–60)		
			**Yes–*N*** **(%)**	**No, too long–*N*** **(%)**	**No, too short–*N*** **(%)**
Are you satisfied with the completion time?	Patients		25 (81)	6 (19)	–
	Parents		109 (84)	20 (15)	1 (1)

#### Discussing PROMs with the clinician

**Fig. 3:**
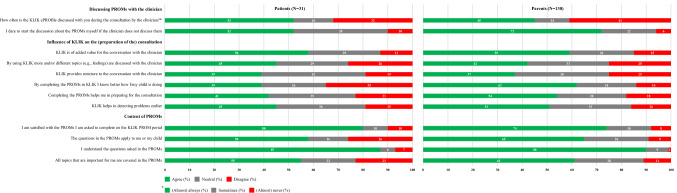
Scores on the domains ‘discussing PROMs with the clinician’, ‘Influence of KLIK on the (preparation of the) consultation’, and ‘content of PROMs’ (patients: *N* = 31, parents: *N* = 130)

About half of the patients and parents indicated that their clinician (almost) always discusses the KLIK ePROfile with them during the consultation (Fig. 3). If the clinician does not discuss the completed PROMs, 52% of the patients and 72% of the parents indicated they dare to start the discussion about PROMs themselves.

#### Influence of KLIK on the (preparation of the) consultation

KLIK is of added value for the conversation with their clinician, according to 58% of the patients and 59% of the parents (Fig. 3). Less than half of the patients and parents indicated that more topics are discussed by using the KLIK PROM portal in comparison with not using the KLIK PROM portal and that the use of KLIK provides more structure to the conversation. Clinicians’ failure to discuss the KLIK ePROfile was a frequently mentioned reason why KLIK has no value during the consultation. More than half of the parents reported that the use of KLIK provides them more insight into the functioning of their child and helps in preparing for the consultation (62% and 54% respectively), in contrast to only 39% and 42% of the patients. Patients indicated that they know very well how they are doing, even without completing a PROM.

Table 5 shows the most important advantages and disadvantages of KLIK, as reported in the open questions. The themes are ranked based on the number of times mentioned by patients and parents in the open-ended questions. Main advantages of KLIK for patients and parents were: *easy to use, clinician is better prepared, patients and parents are better prepared*, and *insight into functioning (of my child)*. Main disadvantages of KLIK for patients and parents were: *not easy to use, irrelevant content of PROMs*, and *takes time*. Eleven patients (35%) and 48 parents (37%) did not experience any disadvantages with using the KLIK PROM portal.

**Table 5:** Advantages and disadvantages of the KLIK PROM portal, mentioned by patients (*N* = 31) and parents (*N* = 130) in the open questions of the evaluation questionnaire

	Examples
**Advantages KLIK PROM portal**	
Easy to use	‘Simple and clear’ ‘It is easy that you can complete questionnaires online at home’
Clinician is better prepared	‘The clinician can see my questions before the appointment at the outpatient clinic’ ‘The clinician is already aware of my child's health situation and can immediately respond to it’
Patient and parents are better prepared	‘It is valuable that you can ask the clinician questions in advance so that you do not forget them’ ‘Subjects are discussed which you normally do not bring up yourself’
Insight into functioning (of my child)	‘KLIK provides insight into how I am doing’ ‘Provides the opportunity to compare the health situation of my child now with the situation just after diagnosis’
**Disadvantages KLIK PROM portal**	
Not easy to use	‘I keep forgetting my password’ ‘Annoying that I get multiple reminders’
Irrelevant content of PROMs	‘Not all questions apply to our situation’ ‘It is boring to complete the same questionnaires every time’
Takes time	‘Completing the questionnaires takes sometimes more time than I hope’ ‘It is a lot of work to complete the questionnaires’

All quotes were translated into English

#### Usability of the KLIK PROM portal

The KLIK PROM portal is easy to use, according to 81% of the patients (13% neutral and 6% disagree) and 74% of the parents (18% neutral, 8% disagree). In addition, 48% of the patients (39% neutral, 12% disagree) and 55% of the parents (36% neutral, 9% disagree) indicated that KLIK has an attractive layout.

#### Content of PROMs

Most patients and parents are satisfied with the PROMs they are asked to complete (Fig. 3). Almost all participants indicated that they understand the questions asked in the PROMs. Reasons why patients and parents are not satisfied with the offered PROMs were that the questions in the PROMs do not apply to them or their child, PROMs are too generic, the different questions are very similar, and the PROMs are too long. Some of the patients and parents felt that the offered PROMs do not cover all topics that are important for them. For example they miss topics like growth, parenting support, and side jobs.

## Discussion

This study provided insight into the experiences of patients and parents with the implementation of PROMs in pediatric clinical practice using the KLIK PROM portal. Overall, patients and parents were satisfied with the use of KLIK. They indicated that KLIK provides insight into the patient’s functioning, helps parents and clinicians in preparing for the consultation, is easy to use, and results in discussion of a broad range of topics (e.g., from disease-specific to psychosocial functioning) during the consultation. However, points of improvement were indicated regarding the content of PROMs, the layout of the KLIK PROM portal, and the discussion of PROMs by the clinician. The results described in this study are in line with previous studies [15, 25, 26].

Although patients and parents responded to the closed question of the evaluation questionnaire that they are generally satisfied with the offered PROMs in KLIK, they mentioned in the focus groups and open-ended questions that the content of PROMs is the most important point of improvement. For example, they indicated that there is repetition in questions, that irrelevant questions are administered, and that the completion time is long, resulting in a burden of completing PROMs. These challenges with PROMs have been mentioned in previous research [16, 35, 36]. To address these challenges, the self-report and proxy-versions of the Patient-Reported Outcomes Measurement Information System (PROMIS^®^) item banks [37–39] were implemented in the KLIK PROM portal in the past year and are currently used in several clinics [16, 40, 41]. The PROMIS item banks each measure a separate construct that can be administered using Computerized Adaptive Testing (CAT). With CAT, questions are presented to patients based on their previous responses. Hence, patients only have to answer a small number of questions per item bank to obtain a reliable score [42] and have to answer less irrelevant questions. Consequently, the burden of completing PROMs can be reduced.

Another difference between the focus groups and the questionnaire was the rating of the ease of use of the KLIK PROM portal. While in the questionnaire the majority of participants indicated that KLIK is easy to use, in the focus groups especially parents had quite some remarks on how the ease of use could be improved. Parents mentioned that an app would be a valuable addition to the KLIK website in order to complete PROMs on your mobile phone. Additionally, they would like an integration of KLIK with the EHR so that appointments are automatically linked to KLIK by which PROMs are directly available. To address these suggestions, we made the KLIK PROM portal adaptable for mobile phone use, and realized a front-end (hybrid) integration with the EHR in 2019. With this integration, clinicians can now view the KLIK ePROfile in the EHR and discuss the PROMs more easily. However, to be able to automatically link the appointments to KLIK, a full integration is necessary, which can hopefully be realized in the future.

A final difference between the focus group and questionnaire outcomes was the satisfaction with the layout of the KLIK PROM portal, which was mainly mentioned as a point of improvement in the questionnaire. Patients and parents indicated that the website looks a bit old-fashioned and could be made more attractive by using visuals. For this reason, the homepage of the KLIK website was upgraded recently. The design of the website was changed (e.g., by using visuals and creating a more professional look). In addition, specific information pages are now available for all KLIK users (pediatric patients, parents, adult patients, and clinicians).

Patients and parents mentioned in both the focus groups as the questionnaires that clinicians often do not discuss PROMs during the consultation. This is worrisome, as patients and parents indicated that this is an important reason why KLIK sometimes has no added value for the consultation which consequently may lead to loss of motivation to complete KLIK PROMs. To improve this discussion rate, several implementation strategies were used. For example, the KLIK expert team revised the KLIK training in which more attention is now paid to the importance of discussing PROMs [43] and this topic is discussed more thoroughly during annual evaluation meetings with clinicians [16], with the goal to increase their knowledge, awareness and confidence in discussing PROMs. Additionally, finding champions for each multidisciplinary team to motivate clinicians to use and discuss KLIK PROMs would be beneficial as this was identified as the most important implementation strategy in two KLIK studies [15, 17]. When clinicians do not discuss the completed PROMs, patients and some parents indicated that they do not dare to bring up for them important themes themselves. To empower patients/parents and increase their self-efficacy, educational videos were developed and made available on the KLIK homepage (article in preparation). In these videos tips and tricks are provided how patients and parents can prepare themselves for the consultation and bring up topics they want to discuss with the clinician.

When comparing this study with the KLIK evaluation study with clinicians [16], similar experiences regarding the KLIK PROM portal were mentioned. For example, insight into patients’ functioning, improved communication, and better preparation of the consultation were positive points they agreed on, and content of PROMs was the most important point of improvement mentioned by both user groups. However, patients/parents and clinicians mentioned a different PROM completion rate. Patients and parents indicated a very high completion rate, whereas clinicians estimated that this completion rate is much lower and that it takes a lot of effort to motivate patients to complete PROMs [16]. A possible reason for this difference might be a bias in the current sample, as only patients and parents that were part of the KLIK panel were invited for participation. These patients/parents might be more assertive in comparison to the other KLIK users, which might have resulted in an overestimation of the PROM completion rate. Therefore, continuous support and explanation about the goal of the use of KLIK remains very important to both user groups.

There are some limitations to this study that should be mentioned. First, there was a low response rate in the evaluation questionnaire (around 20%) which was unexpected as this questionnaire was sent to participants of the KLIK panel (who indicated that they were willing to be invited for research projects). Possible reasons for the low response rate might be that (1) the willingness of patients and parents has changed as participation in the KLIK panel was only asked during registration, (2) patients and parents do not actively use the KLIK PROM portal anymore, or (3) patients and parents might be tired of completing surveys. Second, it was also difficult to motivate patients to participate in the focus groups. This resulted in a small number of participants per patient focus group (2 to 3 participants) with two moderators, which may have influenced the dynamics. Additionally, we noticed that pediatric patients found it very difficult to formulate and express their opinion and needed a lot of guidance which could have led to a bias in the results. Third, we used a self-developed questionnaire which makes comparisons with other evaluation studies difficult. However, other studies also made use of self-conducted questionnaires [44] or adapted questionnaires from prior studies [29–31], as the questions needed to be specific about features of the tool used.

In conclusion, pediatric patients and parents were satisfied with the usability and effect of the KLIK PROM portal in clinical care. KLIK provides them insight into their functioning and helps them to communicate with the clinician. However, some points of improvement were also identified, which are currently being addressed. We now have insight into the experiences of the most important stakeholders (patients/parents and clinicians) of KLIK. In the future it is important to continuously evaluate the use of the KLIK PROM portal with all stakeholders (including adult patients) to match their needs. In this way, we can further optimize and implement the KLIK PROM portal in clinical care with the ultimate goal to improve the quality of care.

## Data Availability

The datasets generated during and/or analyzed during the current study are available from the corresponding author on reasonable request.
