# Correction to: Patients’ and parents’ perspective on the implementation of Patient Reported Outcome Measures in pediatric clinical practice using the KLIK PROM portal

**DOI:** 10.1007/s11136-021-03072-0

**Published:** 2022-01-20

**Authors:** Maud M. van Muilekom, Lorynn Teela, Hedy A. van Oers, Johannes B. van Goudoever, Martha A. Grootenhuis, Lotte Haverman

**Affiliations:** 1 Child and Adolescent Psychiatry & Psychosocial Care, Amsterdam Reproduction and Development, Amsterdam Public Health, Amsterdam UMC, University of Amsterdam, Emma Children’s Hospital, Meibergdreef 9, Amsterdam, The Netherlands; 2 Department of Pediatrics, Amsterdam UMC, University of Amsterdam, Vrije Universiteit Amsterdam, Emma Children’s Hospital, Meibergdreef 9, Amsterdam, The Netherlands; 3 Princess Máxima Center for Pediatric Oncology, Utrecht, The Netherlands; 4 Child and Adolescent Psychiatry & Psychosocial Care, G8-136, Amsterdam UMC, University of Amsterdam, Emma Children’s Hospital, Amsterdam, The Netherlands 22660, 1100 DD

## Correction to: Quality of Life Research 10.1007/s11136-021-02950-x

In the original publication, Fig. 3 was published incorrectly. The correct version of Fig. 3 is provided in this correction.

**Fig. 3:**
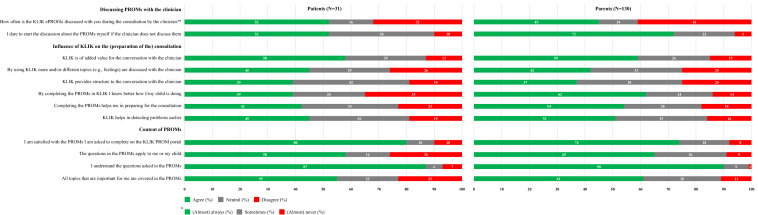
Scores on the domains ‘discussing PROMs with the clinician’, ‘Influence of KLIK on the (preparation of the) consultation’, and ‘content of PROMs’ (patients: *N* = 31, parents: *N* = 130)

The original article has been corrected.

